# Supplementary material for: Content-rich biological network constructed by mining PubMed abstracts
Source: BMC Bioinformatics. 2004 Oct 8;5:147. doi: 10.1186/1471-2105-5-147 (PMC528731; doi:10.1186/1471-2105-5-147)
Supplement: Additional File 2 — The original results of the above study (non-essential files are deleted to keep the file size under the limit set by BMC bioinformatics). [file 1471-2105-5-147-S2.bz2 › chilibotAdditionalFile2/dip20/685ID8980235E18004/html/IHFA_IHFB.html]

 


 **IHFA** and **IHFB** 
  
Found 14 abstracts in PubMed, retrieved 14.  
 

 What does Google say? 
 PDF only 
| .edu only 

---

**Interactive relationship** (e.g. stimulation, inhibition, etc)

**Neutral relationship**- We show that IHF has a very strong preference to bind to  **ihfb**  even when a mutated  **ihfa**  has a better match with the consensus.  Ref: 1829157 Nucleic Acids Res, 1991
- Chimeric proteins between Escherichia coli histone like HU and IHF were constructed by genetic engineering, in which part of the arm region was replaced by the corresponding region of IHF alpha  [ **IHFA** ]  designated as HupANhimA or IHF beta  [ **IHFB** ]  HupANhimD.  Ref: 1387378 Gene, 1992

**Non-interactive relationship** (e.g. studied together, co-existance, homology, etc.)

- The predicted products of himA 100 aa, 11.5 kDa and of himD 94 aa, 10.6 kDa share 77 and 70% identity with IHF alpha  [ **IHFA** ]  and IHF beta  [ **IHFB** ]  of Ec, respectively.  Ref: 7867950 Gene, 1995
- The P. putida  **ihfA**  and  **ihfB**  genes can effectively complement E. coli ihf mutants, suggesting that the P. putida IHF subunits can form functional heterodimers with the IHF subunits of E. coli.  Ref: 8892836 J Bacteriol, 1996
- Also, the increase in expression was more pronounced when the IHF protein was mutated in the  **ihfB**  gene than in the  **ihfA**  gene.  Ref: 9735316 Plasmid, 1998
- alternatively, an alpha helix 2 beta 1 region was replaced by the corresponding region of IHF alpha  [ **IHFA** ]  HupAXhimA or IHF beta  [ **IHFB** ]  HupAXhimD symbols N and X indicate NotI and XhoI junctions.  Ref: 1387378 Gene, 1992
- This contention is supported by deletion analysis of the region and the observation that insertional inactivation of genes that reduce transcription from PcM, clpP ATP protease, himA IHF alpha  [ **IHFA** ] , and himD IHF beta  [ **IHFB** ] , block the LamB phenotype.  Ref: 7494472 Mol Microbiol, 1995
- The  **ihfA**  and  **ihfB**  genes encode polypeptides predicted to be 99 and 93 amino acids long, respectively, and are very similar to integration host factor subunits from other Gram negative bacteria, although phylogenetic analysis indicated that the P. haemolytica sequences are distantly related to those from other bacteria.  Ref: 9011038 FEMS Microbiol Lett, 1997
- In strain MG1655, mutants containing lesions in either the alpha  **ihfA**  or beta  **ihfB**  subunits of IHF show a marked decrease in both FimB 100 fold and FimE 1 00 fold promoted switching.  Ref: 9157242 Mol Microbiol, 1997
- Complementation of  **ihfA**  and  **ihfB**  mutations in E. coli demonstrated that the P. haemolytica gene products form functional heterologous heterodimers.  Ref: 9011038 FEMS Microbiol Lett, 1997
- Within this region two sequences are present  **ihfa**  and  **ihfb**  that match the consensus sequence for IHF binding.  Ref: 1829157 Nucleic Acids Res, 1991
- In this paper we describe the effect of mutations in the non overlapping part of  **ihfa**  and  **ihfb**  on the binding of IHF.  Ref: 1829157 Nucleic Acids Res, 1991
- In attempts to identify subunit specific phenotypes of ihf mutants we analyzed viability, thermoresistance and protein synthesis patterns in  **ihfA**  and  **ihfB**  mutants and their respective parental strains.  Ref: 9924977 Biochimie, 1998
- Protein IHF bound to two sites  **ihfA**  and  **ihfB**  in R388 oriT, as shown by gel retardation and DNase I footprinting analysis.  Ref: 10209739 Mol Microbiol, 1999
- Northern blotting and primer extension analysis defined the tsp for each gene and indicated a disparity in  **ihfA**  and  **ihfB**  message levels over time, with  **ihfB**  mRNA being more abundant throughout the entire growth cycle.  Ref: 9714829 Gene, 1998
- Both the  **ihfA**  and  **ihfB**  genes of P. putida code for 100 amino acid residue polypeptides that are 1 and 6 residues longer than the Escherichia coli IHF subunits, respectively.  Ref: 8892836 J Bacteriol, 1996
- Furthermore, both the  **ihfA**  and  **ihfB**  message levels declined as cells entered the stationary growth phase.  Ref: 9714829 Gene, 1998
- The ihf genes  **ihfA**  and  **ihfB**  were cloned from N. gonorrhoeae through functional complementation of defined Escherichia coli ihf mutants for plating of phage lambda.  Ref: 9714829 Gene, 1998
- Integration host factor IHF is a small heterodimer containing subunits encoded by the unlinked  **ihfA**  himA and  **ihfB**  himD, hip genes.  Ref: 8973312 Gene, 1996
